# Modeling of Short-Pulse Laser Interactions with Monolithic and Porous Silicon Targets with an Atomistic–Continuum Approach

**DOI:** 10.3390/nano13202809

**Published:** 2023-10-23

**Authors:** Maria S. Grigoryeva, Irina A. Kutlubulatova, Stanislav Yu. Lukashenko, Anastasia A. Fronya, Dmitry S. Ivanov, Andrey P. Kanavin, Victor Yu. Timoshenko, Irina N. Zavestovskaya

**Affiliations:** 1 Lebedev Physical Institute of the Russian Academy of Sciences, Leninskiy Prospect 53, 119991 Moscow, Russia; grigorevams@lebedev.ru (M.S.G.); i.kutlubulatova@lebedev.ru (I.A.K.); lukashenko13@mail.ru (S.Y.L.); aafronya@mephi.ru (A.A.F.); kanavinap@lebedev.ru (A.P.K.); zavestovskayain@lebedev.ru (I.N.Z.); 2 Institute of Engineering Physics for Biomedicine (PhysBio Institute), National Research Nuclear University MEPhI (Moscow Engineering Physics Institute), Kashirskoe Shosse 31, 115409 Moscow, Russia; 3 Institute for Analytical Instrumentation of the Russian Academy of Sciences, Rizhsky Prospect, 26, 190103 St. Petersburg, Russia; 4 Faculty of Physics, Lomonosov Moscow State University, Leninskie Gory, 1, 119991 Moscow, Russia; timoshen@physics.msu.ru

**Keywords:** laser ablation of semiconductors, molecular dynamics, atomistic–continuum numerical method, crystal structure, porosity

## Abstract

The acquisition of reliable knowledge about the mechanism of short laser pulse interactions with semiconductor materials is an important step for high-tech technologies towards the development of new electronic devices, the functionalization of material surfaces with predesigned optical properties, and the manufacturing of nanorobots (such as nanoparticles) for bio-medical applications. The laser-induced nanostructuring of semiconductors, however, is a complex phenomenon with several interplaying processes occurring on a wide spatial and temporal scale. In this work, we apply the atomistic–continuum approach for modeling the interaction of an fs-laser pulse with a semiconductor target, using monolithic crystalline silicon (c-Si) and porous silicon (Si). This model addresses the kinetics of non-equilibrium laser-induced phase transitions with atomic resolution via molecular dynamics, whereas the effect of the laser-generated free carriers (electron–hole pairs) is accounted for via the dynamics of their density and temperature. The combined model was applied to study the microscopic mechanism of phase transitions during the laser-induced melting and ablation of monolithic crystalline (c-Si) and porous Si targets in a vacuum. The melting thresholds for the monolithic and porous targets were found to be 0.32 J/cm^2^ and 0.29 J/cm^2^, respectively. The limited heat conduction mechanism and the absence of internal stress accumulation were found to be involved in the processes responsible for the lowering of the melting threshold in the porous target. The results of this modeling were validated by comparing the melting thresholds obtained in the simulations to the experimental values. A difference in the mechanisms of ablation of the c-Si and porous Si targets was considered. Based on the simulation results, a prediction regarding the mechanism of the laser-assisted production of Si nanoparticles with the desired properties is drawn.

## 1. Introduction

The ability of short laser pulses to deposit their energy into a localized region of a material in an extremely short time scale makes them the perfect candidate to be used as the most precise tools in materials surface processing and functionalization [1,2,3]. The laser-induced generation of a single subwavelength [4] or arrays [5,6,7] of structures, plasmon-assisted periodic surface structures formation [8], or the formation of nanoparticles (NPs) due to pulsed laser ablation in liquids (PLAL) [9,10,11,12,13]—all these processes constitute the new IT- [14] and bio-technologies [15]. Additionally, the precise processing of semiconductor materials has become extremely important in the manufacturing of the new generation of electronic devices nowadays. Thus, the generation of laser-induced periodic surface structures (LIPSS) on crystalline silicon (c-Si) can make solar cells surfaces hydrophobic [16]. The laser-induced formation of circular, crisscross, and irregular LIPSS [17,18], as well as twisted nanojet structures [19,20], has revealed their unique properties in enhanced filed microscopy [21] and the generation of antibacterial surfaces [22].

Laser-synthesized Si NPs occupy a special niche associated with applications in biomedicine. They have the necessary purity, excellent biocompatibility, and biodegradability [23,24,25,26]. Nanostructured Si (nanosilicon) luminesces in the visible and near-infrared regions of the spectrum [27,28], which makes it suitable for the development of fluorophores and contrast agents for bio-visualization [29,30,31]. It has been shown that laser-synthesized nanosilicon can serve as an effective sensitizer of radiofrequency hyperthermia for cancer therapy [32]. It is also worth pointing out the use of Si NPs in photothermal therapy [33], drug delivery [34], and carriers of therapeutic radionuclides in nuclear nanomedicine [35,36,37].

In recent decades, PLAL has become one of the leading methods for the efficient production of environmentally friendly NPs, which has shown impressive progress over the past 15 years [13,38,39], due to its versatility and relative cost-effectiveness for the synthesis of various nanomaterials. The performance of the method, however, and the characteristics of the final product depend on many parameters, including the laser wavelength, pulse duration, energy density at the target surface, the irradiated material’s properties, and the composition of the working fluid. Among the most important advantages of the laser ablation technique, in comparison to other methods for obtaining nanoparticles (mechanical, chemical, and so on), is the absence of chemical reagents in solutions. In other words, this method makes it possible to obtain chemically pure colloidal solutions of NPs, free from extraneous impurities and radicals [40]. Also, Si-based NPs can achieve a high porosity that makes their large surface area highly efficient for the bonding of drugs for their subsequent delivery [41,42]. The formation of porous Si NPs in colloidal solutions has also been found to be efficient due to the pulsed laser ablation of porous Si targets in liquids [43,44,45,46].

However, the production of c-Si and porous Si NPs of a given size, morphology, and properties requires the optimization of laser ablation regimes in order to advance the reproducibility of these technologies, and for the possibility of the biomedical application of the obtained nanoparticles, according to their characteristics. In this sense, laser-induced melting and ablation were among the first phenomenon induced in the solid state that established the whole subsequent evolution of irradiated Si targets in vacuum or liquid media. To obtain a reliable knowledge of the underlying physical processes accompanying the laser ablation of Si target c-Si and porous Si and the possibility of their manipulation requires a robust computational model capable of describing a variety of laser-induced phenomena inside the irradiated target, which emerge on a wide spatial and temporal scale.

The molecular dynamics (MD) method seems to be an appropriate tool with which to capture the laser-induced phase transitions. Since it works with atomic precision, the crystal structure and peculiarities of all the crystal defects are taken into account in MD automatically, and the phase transitions themselves are described without any assumption about the character of the process. All of them are given a priori via the utilized interatomic potential function, which can be parametrized in different ways, depending on the required material properties and the experimental conditions under study. Due to their accurate reproduction of the thermophysical properties, for instance, the Stillinger–Webber [47] and Tersoff [48] interatomic potential function parametrizations for Si are among the most frequently utilized.

There are more sophisticated approaches for the description of interatomic potential functions, however. For instance, the bond-weakening phenomena in Si for the description of nonthermal processes under ultrashort laser pulse irradiation was recently addressed [49]. Although possessing low accuracy, this potential is an example of the inclusion of band structure information into the classic MD approach. Some discussions about the necessary conditions that must be fulfilled by such a potential function in order to obtain better accuracy were recently reported in Ref. [50]. Aiming to elaborate on the potentials capable of describing bond-weakening (hardening) processes in materials due to ultrashort laser pulse excitation, more advanced potentials fit to ab initio MD simulations based on density functional theory (DFT) have been recently developed [51,52]. In particular, the interatomic potential as a function of the electronic temperature *U*(*T_e_*) for c-Si [53] has shown promising results for the description of ultrashort-pulse non-thermal melting.

Nevertheless, even the most advanced interatomic potentials for c-Si are still missing an explicit description of the free carriers’ subsystem. Thus, the laser light absorption process, the diffusion of the laser-generated free carriers and their thermal energy transport, and the effect of a strong non-equilibrium between the free carriers and lattice temperatures (induced with an ultrashort laser pulse) are still out of consideration. Discarding the above effects can lead to unphysical confinement of the laser-deposited energy within proximity of the irradiated material’s surface. To overcome this obstacle, in this work the classic MD approach is modified by means of its combination with the description of the free carriers’ temperature and density dynamics in continuum. Such an idea was first realized for metals [54], and combines the advantages of two fundamentally different numerical methods: molecular dynamics (MD) for the description of the kinetics of the non-equilibrium laser-induced phase transformations with atomic resolution, and the two temperature model (TTM) [55], which considers the effect of the free carriers in continuum.

In this work, therefore, we undertake an attempt to advance the understanding of the microscopic mechanism of the ultrashort pulse laser-induced melting and ablation of c-Si and porous Si targets. For this purpose, we modified and utilized our previously proposed combined atomistic–continuum model for Si [56], in the framework of which the kinetics of the non-equilibrium laser-induced phase transition will be studied with atomic precision via the MD method, with the interatomic potential function parametrized according to the Stillinger–Webber potential [45]. On the other hand, the effect of the free carriers generated by the laser pulse will be accounted for in the continuum with the help of the model proposed by van Driel [57]. Similar to the TTM for metals, the proposed models describe the irradiated Si target evolution via the electron–hole carriers’ temperature and density dynamics. The required continuum description of the free carriers’ evolution in Si, however, must account for the non-constant carriers’ density in the conduction band, which makes it a much more advanced, but computationally expansive, method for processing as compared to the well-known TTM. Thus, the required model will account for the absorption of the laser light, the process of the laser-generated carriers’ diffusion and their fast heat conduction, as well as the strong electron–phonon non-equilibrium generated in the irradiated target with an ultrashort laser pulse. The combined model, therefore, unifies the advantages of both the abovementioned MD and continuum descriptions of the evolution of the irradiated Si target. Also, unlike its predecessor [54], the combined model proposed in this work is modified to explicitly account for the target’s porosity, and it is adapted for working with the fluencies of the order of the ablation threshold. The complete description of how the presence of porosity was considered in the numerical algorithm is presented in the supplemental materials included with this work. Thus, we begin our research on the nanoprocessing of Si-based materials for technological and bio-medical applications with an investigation of the microscopic mechanism of ultrashort pulse laser melting and ablation. For that purpose, we perform a series of simulations of ultrashort laser pulse interactions with thick Si targets at the fluencies of melting and ablation thresholds for both monolithic and porous materials. The kinetics of the non-equilibrium laser-induced processes are then extracted with atomic precision.

## 2. Materials and Methods

As compared to the already developed atomistic–continuum MD-TTM methods for metals [58,59], a similar approach for Si requires an additional equation for the density of the free carriers. Thus, in addition to the description of the fast electron heat conduction via the thermal diffusion process and the strong non-equilibrium between the free carriers (conduction band electrons in metals) and phonons, the model for semiconducting Si should take into account the dynamical change in the free carriers’ (the electron–hole pairs for semiconductors) density via the one- or two-photon absorption processes, the Auger recombination, impact ionization, and the carriers’ diffusion process as well [60,61,62,63]. Due to an additional equation for the carriers’ density, the combined model for Si, therefore, is referred to as MD-nTTM (where *n* stands for the carriers’ density). Such an approach was suggested in Refs. [54,64] and, for the first time, allowed for the investigation of the microscopic mechanism of the short laser pulse melting of a Si target. In this work, we use the MD-nTTM model to extract the mechanism of the ultrashort laser pulse interaction with free-standing, thick Si targets. In particular, the melting thresholds of the monolithic and porous targets will be found and compared to their experimental values. Also, the mechanism of the short laser pulse ablation of c-Si and porous Si will be investigated.

The description of the free carriers’ temperature, *T_e_*, dynamics in Si, as compared to TTM, is accompanied by an additional equation for the density of the free carriers, *n_e_*, which was proposed by van Driel and Chen [55,65]. The corresponding system of coupled equations for the light intensity attenuation, free carrier density, energy flux, and temperatures of both the carriers and lattice can be written in the following way:

(1) $$\begin{array}{l} \frac{dI\left(z,t\right)}{dz}=-\left(\alpha +\alpha_{FCA}\left(\varepsilon \left(N_{e}\right)\right)\right)I\left(z,t\right)-\beta I^{2}\left(z,t\right)\\ \frac{\partial n_{e}}{\partial t}=\frac{\alpha I\left(z,t\right)}{h\nu }+\frac{\beta I^{2}\left(z,t\right)}{2h\nu }+\delta n_{e}-\gamma N_{e}^{3}-\nabla \cdot \vec{J}(n_{e},T_{e},E_{g})\\ \frac{\partial U_{e}(N_{e},T_{e},E_{g})}{\partial t}=\left(\alpha +\alpha_{FCA}\left(\varepsilon \left(N_{e}\right)\right)\right)I\left(z,t\right)+\beta I^{2}\left(z,t\right)\\ -\nabla \cdot \vec{W}(N_{e},T_{e},E_{g})-\frac{C_{e-h}(N_{e},T_{e},E_{g})}{\tau_{e}}\left(T_{e}-T_{l}\right)\\ C_{l}\frac{\partial T_{l}}{\partial t}=\vec{\nabla }\cdot \left(\kappa \vec{\nabla }T_{l}\right)+\frac{C_{e-h}\left(N_{e},T_{e},E_{g}\right)}{\tau_{e}}\left(T_{e}-T_{l}\right)\to \to m_{i}\frac{d{\vec{r}}_{i}}{dt^{2}}=-\sum_{j=1,N}^{\;}\vec{\nabla }U\left({\vec{r}}_{ij},T_{e}\right)+\xi m_{i}{\vec{V}}_{i}^{T} \end{array}$$

where *α, β, δ,* and *γ* and are the coefficients of the one- and two-phonon absorption, impact ionization, and Auger recombination, respectively; *I* is the excitation intensity; and $\alpha_{FCA}$ is the free carrier absorption coefficient. The systems of the free carriers (electron–hole pairs) and the lattice interact via the electron–phonon coupling, defined here as the ratio of the free carrier’s heat capacity, *C_e-h_*, and the electron–phonon energy relaxation time, *τ_e_*. It is noteworthy that the energy flux, *W,* in Equation (1) includes both the carrier’s flux, *J*, with the diffusion coefficient, *D,* where the heat diffusion is defined via the conductivities of both the electrons and holes as *k_e_* and *k_h_*:

(2) $$\begin{array}{l} \vec{W}=\left\{E_{g}+2k_{B}T_{e}\left[H_{0}^{1}\left(\eta_{e}\right)+H_{0}^{1}\left(\eta_{h}\right)\right]\right\}\vec{J}-\left(k_{e}+k_{h}\right)\nabla T_{e}\\ \vec{J}=-D\left\{\nabla n_{e}+\frac{n_{e}}{k_{B}T_{e}}{\left[H_{-\frac{1}{2}}^{\frac{1}{2}}\left(\eta_{e}\right)+H_{-\frac{1}{2}}^{\frac{1}{2}}\left(\eta_{h}\right)\right]}^{-1}\nabla E_{g}+\frac{n_{e}}{T_{e}}\left[2\frac{H_{0}^{1}\left(\eta_{e}\right)+H_{0}^{1}\left(\eta_{h}\right)}{H_{-\frac{1}{2}}^{\frac{1}{2}}\left(\eta_{e}\right)+H_{-\frac{1}{2}}^{\frac{1}{2}}\left(\eta_{h}\right)}-\frac{3}{2}\right]\nabla T_{e}\right\}\\ D=\frac{k_{b}T_{e}}{q_{e}}\frac{\mu_{e}\mu_{h}H_{\frac{1}{2}}^{0}\left(\eta_{e}\right)H_{\frac{1}{2}}^{0}\left(\eta_{h}\right)}{\mu_{e}H_{\frac{1}{2}}^{0}\left(\eta_{e}\right)+\mu_{h}H_{\frac{1}{2}}^{0}\left(\eta_{h}\right)}\left[H_{-\frac{1}{2}}^{\frac{1}{2}}\left(\eta_{e}\right)+H_{-\frac{1}{2}}^{\frac{1}{2}}\left(\eta_{h}\right)\right] \end{array}$$

where *μ_e_* and *μ_h_* are the mobilities of the electrons and holes, respectively, *H_j_^i^*(*η_c_*) *= F^i^(η_c_)/F^j^(η_c_)* and *F^j^(η_c_)* are the Fermi–Dirac integrals of order “*i*” for the carriers with the reduced chemical potentials *η_e_ = (E_F_* − *E_c_)/kT_e_* and *η_h_ = (E_V_* − *E_F_)/kT_e_* of the corresponding electrons and holes, with respect to their quasi-Fermi levels of conduction and valence bands. Finally, the total energy of the free carriers, *U_e_*, consists of both the kinetic energy and the potential energy inputs, which accounts for the energy gap, *E_g_*, variations as well [63]:

(3) $$U_{e}=N_{e}E_{g}\left(N_{e},T_{e}\right)+\frac{3}{2}N_{e}k_{b}T_{e}\left[H_{1/2}^{3/2}\left(\eta_{e}\right)+H_{1/2}^{3/2}\left(\eta_{h}\right)\right].$$

**Figure 1:**
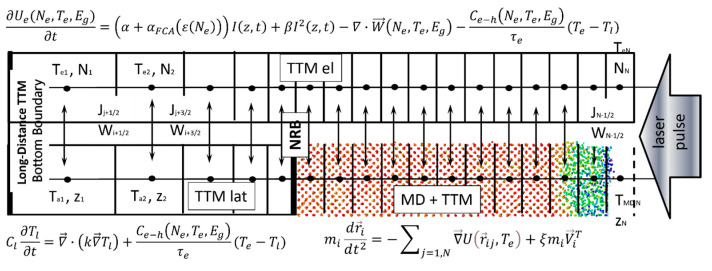
Schematic representation of the computational cell implementing the MD-nTTM model, given by Equation (1). The system of free carriers is discretized into equally spaced cells, *Z_i_*, within the geometry of the MD domain, and the geometrical progression of their position is applied below the non-reflecting boundary conditions (NRB). Below NRB, the nTTM model is solved for both the electron–holes and lattice, providing the heat flux up to a few tens of microns below the surface. Temperature and density values are prescribed for each *Z_i_* position, whereas the carrier’s current, *J_i+_*_1/2_, and energy flux, *W_i+_*_1/2_, are determined between each pair of cells (on their borders). The MD is solved within a depth of a few hundred nm below the surface, where the most intensive processes (including the non-equilibrium phase transitions) may occur. Each cell in the electronic system corresponds to the same volume in the MD domain. The energy exchange between hot carriers and MD particles is accounted for via scaling of the thermal velocities ***V^T^*** = ***V_i_***−***V^c^***, where ***V^c^*** is the velocity of the center of mass of cell ***i***. The MD-nTTM model [54], therefore, is similar to that for metals [52], but with an additional differential equation standing for the density of the free carriers, *N_e_*.

While the description of a number of parameters, using the above continuum model, can vary and be justified either by the experimental measurements [66] or the theoretical calculations [58,67], the strategy for the development of the combined atomistic–continuum model for a semiconductor using the example of Si remains the same as that developed for metals. Namely, we replace the last equation in Equation (1) for lattice temperature with the MD integrations, and the interatomic potential *U* can be used in the classic Stillinger–Webber parametrization [45]. In the future, however, it could be a function of electronic temperature and the free carriers’ density [49]. Finally, it is worth mentioning that unlike a similar model for c-Si published in Ref. [62], where a non-degenerate model based on the Boltzmann distribution of the electron–hole pairs was assumed (indicating negligible excitation), the complete description of the laser-generated free carriers’ dynamics via the Fermi–Dirac functions was applied in this work, and resulted in the applicability of the combined MD-nTTM model for the investigation of not only laser melting [54], but the ablation processes of Si as well. Moreover, as compared to the previously suggested models [54,62,68,69], the approach presented here explicitly accounts for the density change along the target’s volume (due to pre-existing porosity or the development of internal voids due to the ongoing processes of foaming and spallation) and the dynamical change of the carries’ density during the target’s evolution. Although this change is accounted for in the model in a straightforward way via the simple linear scaling of the carriers’ properties, using Equation (1) this approach, for the first time, provides the numerical description of the porous target’s evolution due to an fs-laser irradiation. To avoid further overloading of the already complicated description of the free carriers’ dynamics given by Equation (1), we give a detailed description of the varying density (porosity) inclusion in the supplemental materials. It is also worth mentioning here that, with an appropriate modification of the free carriers’ properties in Equation (1), the applied model can be reasonably extended to other semiconductor materials. The combined MD-nTTM model, which is suitable for the investigation of the microscopic mechanism of ultrashort-pulse laser-induced melting and ablation of monolithic and porous targets, is schematically presented in Figure 1.

From the point of view of the practical application of the MD-nTTM model for c-Si and porous Si on an experimental scale, however, there are several significant implications, some of which are yet to be solved. First, the use of a simple and easy-to-implement (especially for MPI multiprocessing) explicit finite difference scheme for finding the energy of free carriers in Eq. 1 requires the use of an extremely shallow time step: ~10^−22^–10^−24^ s, as compared to ~10^−17^ s for metals with similar spatial discretization. In turn, this leads to the very low efficiency of the combined MD-nTTM model and the impossibility of its utilization on an experimental scale. The first problem was solved in Ref. [70], by applying the implicit numerical Crank–Nicolson scheme in 1D, which made it possible to increase the integration time step by 6–7 orders of magnitude for spatial discretization in the range of 1–5 nm. However, due to a major sequential part of the Crank–Nicolson algorithm, this approach is, in practice, not amenable to parallelization. Thus, the presented MD-nTTM model is still limited to 1D conduction, leaving the possibility of simulations of laser–Si interaction on an experimental scale outside the bounds of consideration for the moment. The efficient modification of the Crank–Nicolson algorithm for parallelization in 3D was recently suggested by [71], and could be utilized in the future for MPI processing to perform simulations on the nanostructuring of Si material on an experimental scale.

It should be noted here that the method of porosity imitation chosen by our computational approach is limited to the lateral size of the computational cell, which is due to the high computational cost for the order of just a few nm. Apart from the origin of pores of that small a size, this approximation assumes to account for the near-field effect during the laser light absorption process, which was omitted in the present calculations. Thus, the inclusion of porosity in this model is only limited by the resulting mechanical properties of the Si sample, with a corresponding decrease in its conductivity properties. As mentioned above, this decrease in our work was accounted for by a simple linear approximation; however, more advanced approaches were recently demonstrated for metals [72,73]. The detailed numerical algorithm of the proposed MD-nTTM model in 1D, accounting for the preexisting target’s porosity, can be found in the supplemental materials.

## 3. Results and Discussion

Using the MD-nTTM model, the melting threshold of 1000 nm thick Si was determined using 270 fs laser pulse irradiation at a wavelength of 800 nm. Since the melting threshold is one of the quantitative results of this work, we determined it as the energy input density resulting in surface melting up to a depth of a few nm maximum. This assumes the fast advance of the melting front to its maximum depth, with a slow process over a longer time scale via the heterogeneous mechanism of resolidification (with a slow propagation of the solid–liquid interface, in other words). For this purpose, we performed a series of modelings at selected incident fluencies of 0.24–0.32 J/cm^2^. The corresponding thresholds for the monolithic and porous targets could differ, not only because of the physical properties changes of the materials (density), but also due to the reduced conductivity properties of the free carriers in the porous target. For the ablation threshold, the sequence of simulations was performed for a range of incident fluences of 1–10 J/cm^2^. The initial c-Si sample with dimensions of 2.6 nm × 2.6 nm × 1000 nm was created and equilibrated at normal conditions (T = 300 K, P = 0 GPa). Assuming that we were modeling the material at the laser spot center, periodic boundary conditions were applied in the direction perpendicular to the laser pulse propagation, whereas free boundaries were applied in the direction of the pulse. The sample thickness (~1000 nm) was chosen as large enough to accommodate the laser-induced processes and avoid the sample-thickness effect on the modeling results. The latter were checked for thicker targets. Finally, a porosity of ~33% by volume was introduced in a straightforward way by cutting equally spaced empty spheres of 2 nm in diameter along the whole sample’s central axis. By doing so, we intended to study solely the porosity effect without any assumption about its origin.

The value of the absorbed laser energy was calculated from the equation for the density of the free carriers (the second line in Equation (1)), where the reflected signal was calculated according to the Drude model of light’s interaction with free carriers. It should be noted that the latter resulted in a significant increase in the integral value of the reflectivity function, from 0.53 to 0.96, and the fluence varied from 0.32 to 10 J/cm^2^, respectively. At high incident fluencies, this corresponds to an increase in the number of free charge carriers in the subsurface layer of the irradiated target, and the asymptotic approach of the dielectric function of the target to its values in metals.

**Figure 2:**
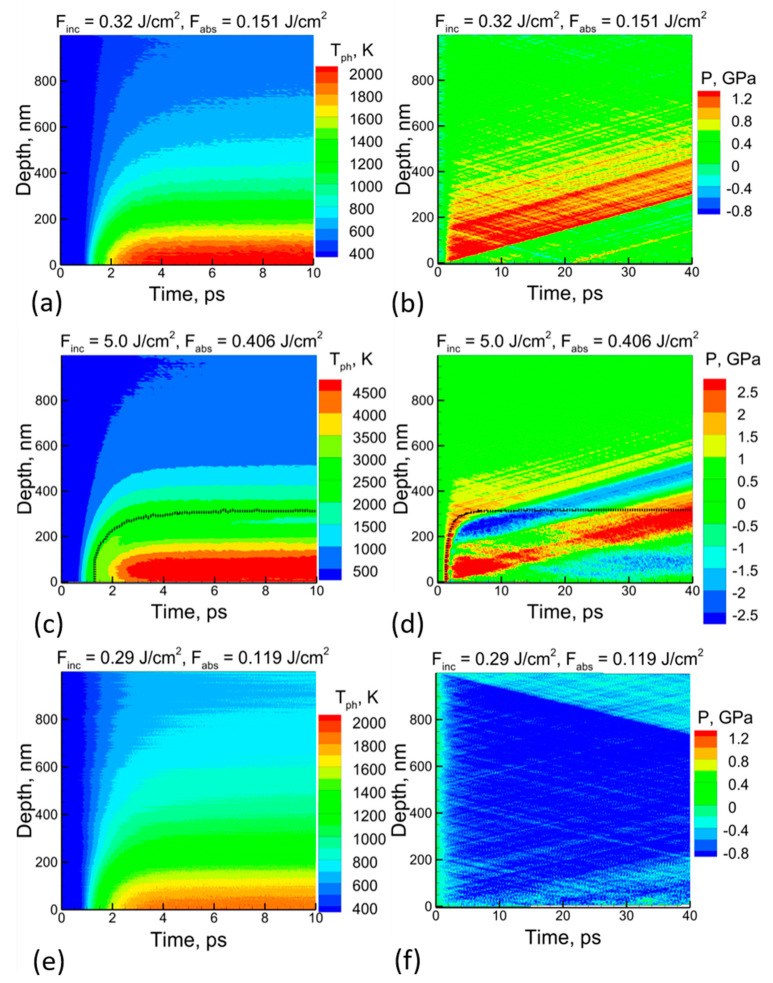
(**a**) Spatial–temporal field of the lattice temperature and (**b**) pressure evolutions inside the c-Si target upon 270 fs pulse irradiation at 800 nm with an incident fluence of 0.32 J/cm^2^. The (**c**) spatial–temporal field of the lattice temperature and (**d**) pressure evolutions inside the c-Si target after irradiation with a 270 fs pulse at a wavelength of 800 nm and an incident fluence of 5.0 J/cm^2^. (**e**) Spatial–temporal field of the lattice temperature and (**f**) pressure evolutions inside the porous (33% by volume) Si target after irradiation with a 270 fs pulse at a wavelength of 800 nm with an incident fluence of 0.29 J/cm^2^. In all pictures the laser pulse is directed from the bottom. The dotted line in (**c**,**d**) indicates the solid–liquid interface. Additional macroscopic data evolution (the electron–hole temperature and the density of free carriers) obtained during the simulation procedure, is given in the supplementary section; see Figure S1.

The results of the modeling of the 270 fs laser pulse interaction with c-Si are shown in Figure 2 as the spatial–temporal lattice temperature (a) and pressure (b) evolution inside the target. The laser pulse was directed from the bottom upward. The choice of the irradiation wavelength (800 nm) was justified due to the existence of a sufficient amount of published theoretical and experimental data for their use in the parametrization of the free carriers’ subsystem. The MD-nTTM method, however, is applicable to other wavelengths as well, with appropriate modifications to the free carriers’ parameters description, as given by Equations (1) and (2).

**Figure 3:**
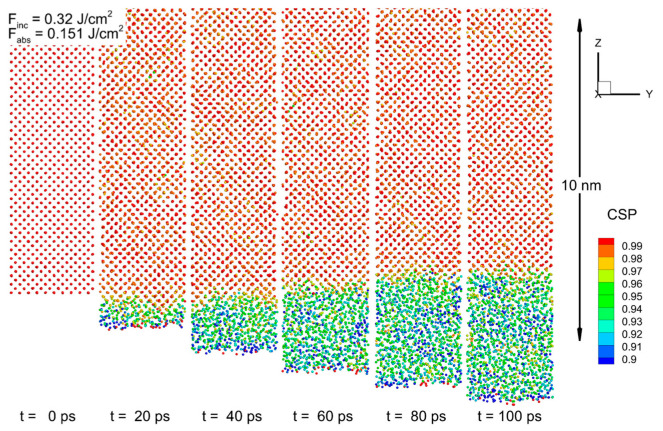
Atomic configurations of Si near its surface (<10 nm) for times of 0, 20, 40, 60, 80, and 100 ps after 270 fs laser pulse irradiation at a wavelength of 800 nm, with an incident fluence of 0.32 J/cm^2^. The color of atoms corresponds to their central symmetry parameter (CSP) for determination of their local order, with empirically chosen criterion between the solid (CSP > 0.97) and the liquid (CSP < 0.97) phases. The laser pulse is directed from the bottom.

Next, the MD-nTTM model was applied to investigate the microscopic mechanisms. Figure 2a shows that at a time of 10 ps after the pulse, a temperature value of more than 1750 K (exceeding the value of the equilibrium melting point of 1683 K) was reached at a depth of up to 100 nm below the surface. However, the nucleation of the liquid phase starting from the surface was detected only at 20 ps. The subsequent advance of the solid–liquid interface at later times did not reach more than 3 nm. The progress of the heterogeneous melting front is illustrated in Figure 3, which shows the atomic configurations of the near-surface layer of the irradiated target for several time values. Moreover, at a rather similar lattice temperature, the values obtained for an incident fluence of 0.3 J/cm^2^ (with the melting point exceeded at a depth of up to 100 nm as well) show that liquid phase nucleation does not occur. The following conclusions, therefore, can be drawn: (1) the melting threshold of the c-Si target found from the simulation with the MD-nTTM model corresponds to a value close to 0.32 J/cm^2^; and (2) the classic thermodynamic approach (based on Gibbs theory or experimentally obtained phase diagrams (obtained under equilibrium conditions)) cannot be applied to the interpretation of experimental data on the short-pulse laser melting of Si. Namely, since the laser-induced phase transition (melting) occurs under non-equilibrium conditions, the classic nucleation theory does not take into account the kinetics of the short laser pulse melting process; thus, missing the amount of energy stored in the irradiated target’s mechanics (acoustical vibrations), and underestimating its threshold value. Thus, the developed numerical approach implementing the MD-nTTM model is a promising method for revealing the kinetics of the microscopic melting mechanisms of irradiated Si targets, since it accounts for the dynamically changing target’s thermodynamic parameters: pressure, temperature, and density.

**Figure 4:**
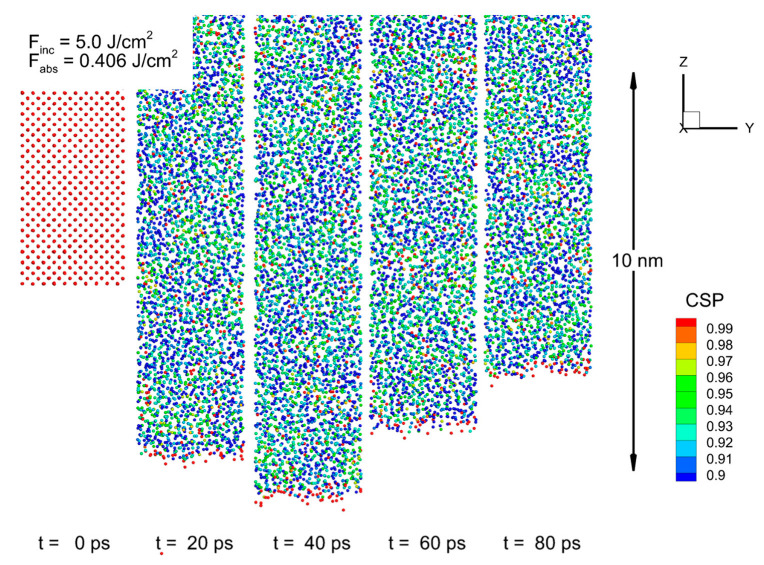
Atomic configurations of Si target near its surface (<10 nm) are shown for the times of 0, 20, 40, 60, and 80 ps after 270 fs laser pulse irradiation at a wavelength of 800 nm, with an incident fluence of 5 J/cm^2^. The color of atoms corresponds to their CSP for determination of their local order, with empirically chosen criterion between the solid (CSP > 0.97) and the liquid (CSP < 0.97) phases of the material. The laser pulse is directed from the bottom.

The laser ablation of the Si targets was conducted for the selected fluencies of 1, 3, 5, 7, and 10 J/cm^2^ at the same pulse duration of 270 fs. The sequence of atomic configurations for the case of 5 J/cm^2^ are shown in Figure 4. Although the silicon surface is completely melted, it can be seen that, apart from a moderate evaporation (red particles), there is no removal of material by the spallation mechanism (in large chunks and droplets, or entire layers) [74]. The reasons for such a weak response by the target to a rather large energy input of short duration (a much shorter pulse length than the characteristic electron–phonon equilibration time of *τ_e-ph_* ~2–5 ps) can be understood by considering the contour plots evolutions of the lattice temperature and pressure inside the irradiated target, as shown in Figure 2c,d.

It has been experimentally established that the thermal expansion of irradiated Si targets is significantly lower than that for metals. This also corresponds to our theoretical calculations, where the linear coefficient of the thermal expansion of Si, given by the Stillinger–Weber potential, was found to be 2.6 × 10^−6^ 1/K, which is an order of magnitude smaller than its value for Al, for instance (~25.5 × 10^−6^ 1/K) [54]. As a result, during the process of electron–phonon interaction and laser-deposited energy transfer from the hot electrons to the lattice, even under conditions of extremely rapid heating, there is no significant accumulation of internal stresses in the pre-surface layer of the irradiated target upon reaching electron–phonon equilibrium. The highest value for the internal stresses of ~2 GPa is an order of magnitude less than that of metal targets (~20 GPa) under similar irradiation conditions. This fact is reflected in Figure 2d, as a moderate acoustic relaxation of the target. But, it was namely the relaxation of the laser-induced pressure process, which was identified for metals as the main driving process of ablation via the spallation mechanism [72].

Second, unlike metals, which mainly have a close-packed crystal structure (fcc or bcc), the crystalline, semiconducting Si is classified as an open-type lattice (an opened diamond), which in turn leads to a negative melting volume of −7.5% (+5% for metals). Thus, even moderate internal stresses inside the Si volume significantly weaken the energy barrier for the nucleation of the liquid phase. Importantly, the accumulation of internal stresses (due to rapid heating) in the fcc crystals and their subsequent relaxation is a key factor for spallation, but the open-type crystal lattice, due to the negative volume of melting, instantly removes any accumulated internal stresses upon phase transition (melting). This suppresses the unloading (rarefaction) pressure wave and, therefore, suppresses the ablation via the spallation mechanism, as was observed in the case of metals [72]. This is also seen in Figure 2d, where at a depth of ~200 nm by ~2 ps the onset of internal stresses accumulation quickly reverts to a negative pressure due to homogeneous melting, thus relaxing the generation of a rarefaction (unloading) acoustic wave.

Figure 2c also reveals that the new phase nucleation that occurs by ~2 ps causes a sharp drop in temperature due to the thermal energy’s transfer into the latent heat of fusion. Further heating of the target due to the electron–phonon coupling results in a new lattice temperature increase, up to a level of ~5100 K (the critical point for Si material is *T_c_*_r_ = 5160 K). For higher temperatures then, the mechanism for the ablation of the irradiated target can have the character of a phase explosion. The onset of this process can be seen from the atomic configurations in Figure 4, where the lighter (higher potential energy) atoms start forming the nuclei of vaporization (boiling). Some amount of vapor Si atoms was also observed at an incident fluence of 5 J/cm^2^. Note that the ablation mechanism through a phase explosion is accompanied by the extraction of a large amount of vapor, which is an advantageous substance for its subsequent condensation (in a liquid medium) into regular-shaped NPs of a single and fine fraction, which are suitable for bio-medical applications [75,76]. It was identified and confirmed by other theoretical groups [57,58,59] that the formation of NPs due to PLAL is caused by two mechanisms: the nucleation of vapor into small NPs and clusters of spherical shape (the fine fraction of NPs distribution), and the decomposition of the Rayleigh–Taylor instabilities onto large droplets with rough shape (the coarse fraction of NPs distribution). While the first mechanism for NPs formation is a consequence of the target’s ablation process via explosive boiling, the second input has its origin in the irradiated target’s ablation process via the spallation mechanism. Namely, the unloading pressure wave that propagates through the excited volume establishes a hydrodynamic motion in the molten part of the materials, which causes the motion of the metal–water interface as well during the PLAL experiment. Eventually, since metal and water have different densities, the interface motion causes the development of Rayleigh–Taylor instabilities.

What is important for c-Si, since the relaxation of the laser-induced stresses is suppressed by the melting process, is that we do not expect the active hydrodynamic motion in the molten part of the material, nor do we expect ablation via the spallation mechanism either. This means that no Rayleigh–Taylor instabilities develop during the ablation of Si in liquids, which leaves the nucleation of vapor from the explosive boiling mechanism as the only mechanism for the formation of Si NPs due to PLAL. While the latter is yet to be confirmed by our consecutive simulations, this prediction is of extreme importance and a very promising step towards the laser-assisted generation of NPs with predesigned properties.

**Figure 5:**
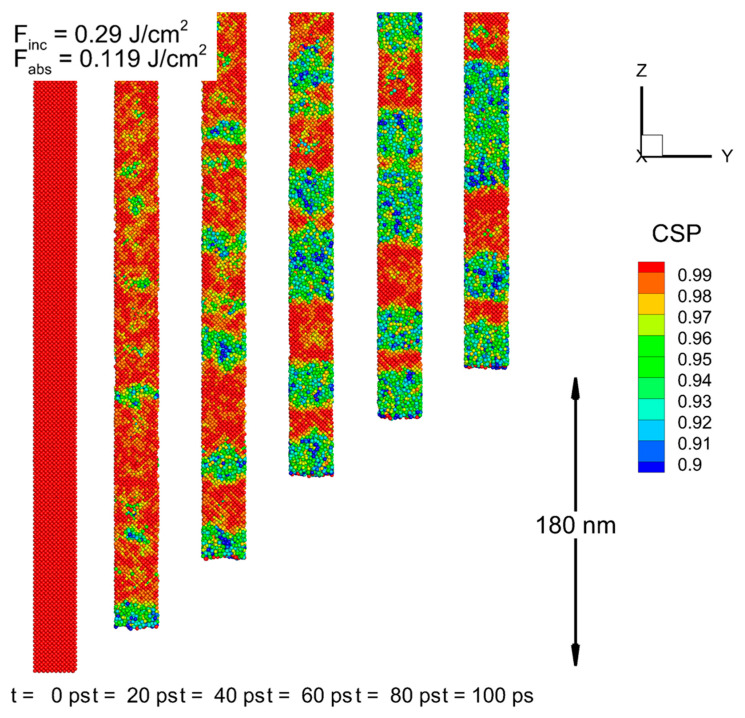
Atomic configurations of porose (33%) Si target are shown up to a depth of 300 nm for selected times of 0, 20, 40, 60, 80, and 100 ps after 270 fs laser pulse irradiation at a wavelength of 800 nm, with an incident fluence of 0.29 J/cm^2^. The color of atoms corresponds to CSP for determination of the local atomic order, with empirically chosen criterion between the solid (CSP > 0.97) and the liquid (CSP < 0.97) phases of the material. The laser pulse is directed from the bottom.

In contrast to the c-Si target, a different evolution of the irradiated target is observed if it has a porosity of 33% by volume, as shown in Figure 5. The presence of internal pores decreases the melting threshold value to 0.29 J/cm^2^, and a significant reduction in volume is observed upon the onset of melting. As indicated above, for the case of a c-Si target, the melting of the pre-surface region occurs after 20 ps whereas, for the porous target, the melting has already begun at 5 ps. This is a consequence of heat accumulation (thermal confinement) due to the limited carriers’ diffusion and thermal heat transport in the pre-surface layer. Also, the presence of pores increases the free surface area, increasing, therefore, the possibility for nucleation of melting via the heterogeneous mechanism.

Figure 2e,f shows the contour plots for lattice temperature and pressure inside the irradiated porous target at a fluence of 0.29 J/cm^2^ (~10% less than the melting threshold value for the c-Si target). As compared to the monolithic target, the accumulation of internal stresses inside the porous one does not take place at all, due to the instant relaxation of any stresses inside the pores. Such an effect was also observed in the investigation of the ablation mechanism of porous metallic targets [74]. Namely, any possible excess of internal stresses due to rapid heating instantly relaxes with the speed of sound in the closest pores. As a result, the activation of compressive–tensile pressure waves is completely suppressed in the case of a porous Si target, as shown in Figure 2f. Consequently, when the laser-induced melting finally occurs, the moderate negative pressure appears due to the negative volume of melting and due to the surface tension forces trying to collapse the internal pores. Such collapse results in the pre-surface layer shrinking due to the motion of the molten material towards the empty volumes, as shown in Figure 5. The adaption of the irradiated porous material to new thermal conditions can also be observed as a moderate pressure wave, propagating from the back surface of the target volume, although this part of the target remains in the solid state. A similar picture was observed during the simulation of a 270 fs laser–Si interaction at a fluence of 5 J/cm^2^ with the porous target. The increased amount of vapor (as compared to the simulation on the c-Si target under the same irradiation conditions) indicated a lower ablation threshold as compared to that of the c-Si target.

It is interesting to note that a qualitatively similar effect of the lowering of the melting threshold was observed experimentally for an ns-pulsed excimer laser irradiation of electrochemically prepared porous Si layers with a porosity of 45% or greater; it was related to both lower thermal conductivity and the greater freedom of the surface atom vibrations in porous Si [77]. Also, Si nanowire arrays with a total porosity of about 60%, under irradiation in a vacuum with 300 ps laser pulses with a wavelength of 805 nm, were found to exhibit 2–3 times lower values for the melting threshold when compared to the corresponding flat Si substrate [78]. Simulations of the fs-ablation of c-Si and porous Si targets using the classic MD method demonstrated a decrease in the ablation threshold for a Si target with porosity above 50%; this effect was explained by the large contribution of the surface and the weakening of the average binding energy of Si atoms on the open surfaces of the pores [79]. However, the microscopic mechanisms by which the threshold lowering occurs in porous semiconducting materials were not addressed in Refs. [45,77,79].

The revealed moderate dependence of the melting threshold on porosity under fs-laser irradiation can be qualitatively compared to the available experimental data, which were obtained for porous Si with a porosity above 45% under laser irradiation with ps and ns pulses, and where the strong restriction of thermal diffusion in the porous material, in comparison to the monolithic one, played the major role [41,42,43,75,76]. According to the experimental data of Ref. [43], the ablation threshold for porous Si under laser irradiation with 20 ps pulses at a wavelength of 1064 nm depended strongly on the targets’ porosity and the surrounding medium (air, water, or ethanol); it was about 0.18 J/cm^2^ for microporous (pore diameter below 2 nm) Si, which was seven times lower than that for the c-Si target subjected to the same PLAL in water.

The examination of the microscopic mechanism of the laser-induced melting of c-Si and porous Si targets also sheds a light on the relative contribution of the different conditions of the laser-melting experiments. Thus, one could expect that a drop in density of the irradiated target by 33% would result in a corresponding decrease in the melting threshold under the equilibrium conditions. This decrease, however, was limited to 10% only, which we associated with the crystal structure of the Si material. As indicated above, the opened-diamond crystal structure is destabilized under an excess of pressure, so the laser-induced stresses facilitated the nucleation of the liquid phase. The porous material, in this case, did not provide the conditions for internal stresses accumulation, and their instant relaxation compensated for the sustainability of the porous Si target against the onset of melting. This resulted in a not-as-strong decrease in the melting threshold as one could have expected, based on the material density change. This finding, however, cannot reliably address the effect of the free carriers’ conductivity change in the porous material. However, one could possibly expect the relative input to result in the decrease in the melting threshold as well.

Finally, the utilization of the developed MD-nTTM model made it possible to reveal the microscopic mechanisms of melting and the ablation mechanisms of monolithic and porous targets by ultrashort laser pulses. It was found that, in contrast to metals, where two mechanisms of laser ablation are observed, phase explosion (explosive boiling) and spallation [72], the latter is rather suppressed in the case of silicon targets. This finding supports a number of the experimental observations and matches other theoretical results, assuming that the ablation mechanism of Si can have the character of explosive boiling and/or Coulomb explosion (not considered in this work) [80,81,82]. Moreover, in the case of a porous target, any accumulation of internal stresses is completely absent, independent of the pulse duration and the energy input (fluence). This makes it possible to draw the following prediction: the use of ultrashort laser pulses at sufficiently large fluencies (above the ablation threshold) during PLAL can promote the fabrication of fine Si NPs with a narrow size distribution. The shape of such NPs is expected to be spherical, since their nucleation from a vapor phase in liquid media is the only possible mechanism for their formation, and the energy input (the incident fluence) is the key parameter responsible for the average size and morphology (monolithic/porous) of the obtained NPs.

## 4. Conclusions

The MD-nTTM model was applied to study the microscopic mechanism of ultrashort pulse laser melting and ablation of c-Si and porous Si targets. The advantage of the model is its ability to consider the kinetics of non-equilibrium phase transitions with atomic precision and, at the same time, to account for the effect of free carriers (the electron–hole pairs). The model allows the description of the absorption of laser pulse irradiation, the diffusion of the excited free carriers (electron–hole pairs), the fast free carriers’ heat diffusion, and lattice heating due to the electron–phonon energy exchange during the relaxation time of 2–5 ps. Thus, the applied model makes it possible to accurately estimate the threshold values of phase transitions of the irradiated Si targets.

The results of the modeling revealed that the kinetics of the melting and ablation of the Si target is very different from its realization in metal targets. Opposite to metals, the increase in pressure in silicon (due to fast laser heating) destabilizes the crystal structure and promotes the initiation of a phase transition (melting) by the homogeneous mechanism. This difference originates from the opened-diamond crystal structure of the Si targets. Subsequently, and especially for the case of the porous Si targets, the ablation mechanism must have the character of a phase explosion due to the achievement of the critical value of the material’s temperature (>5000 K). The melting and ablation thresholds for the c-Si targets were identified as 0.32 J/cm^2^ and ~5.0 J/cm^2^, respectively. The corresponding input into the decrease in the melting threshold due to the drop in density and the effect of the stresses accumulation in a porous target with an opened-diamond crystal structure was discussed. Also, for the porous Si target, the heat flow becomes weaker due to the confinement of the free carriers and the suppression of the heat diffusion processes. The porous morphology of the Si targets, therefore, leads to more efficient heat accumulation in the pre-surface region and, as a result, the melting threshold has a lower value of 0.29 J/cm^2^. Finally, the obtained results not only reveal the microscopic mechanism of the laser-induced melting and ablation processes for the purpose of nanostructuring Si surfaces, but also can be used in the development of a methodology for the laser-assisted production of colloidal solutions of Si NPs, with a controlled size and size distribution for biomedical applications.

## Data Availability

The performed simulation results and obtained data are available by request.

## Supplementary Materials

The following supporting information can be downloaded at: https://www.mdpi.com/article/10.3390/nano13202809/s1. Numerical algorithm implementing the conservative form of the nTTM model. Temporal-spatial evolution fields of free carriers’ density and temperature for low and high fluence regimes for c-Si and porous Si targets. Numerical algorithm of nTTM model with inclusion of porosity description.
