# Supplementary material for: Modeling of Short-Pulse Laser Interactions with Monolithic and Porous Silicon Targets with an Atomistic–Continuum Approach
Source: Nanomaterials (Basel). 2023 Oct 23;13(20):2809. doi: 10.3390/nano13202809 (PMC10609206; doi:10.3390/nano13202809)
Supplement: Supplementary file 1 [file nanomaterials-13-02809-s001.zip › nanomaterials-2637326-supplementary.pdf]

Supplementary Materials: Numerical algorithm implementing the conservative form of the nTTM model.

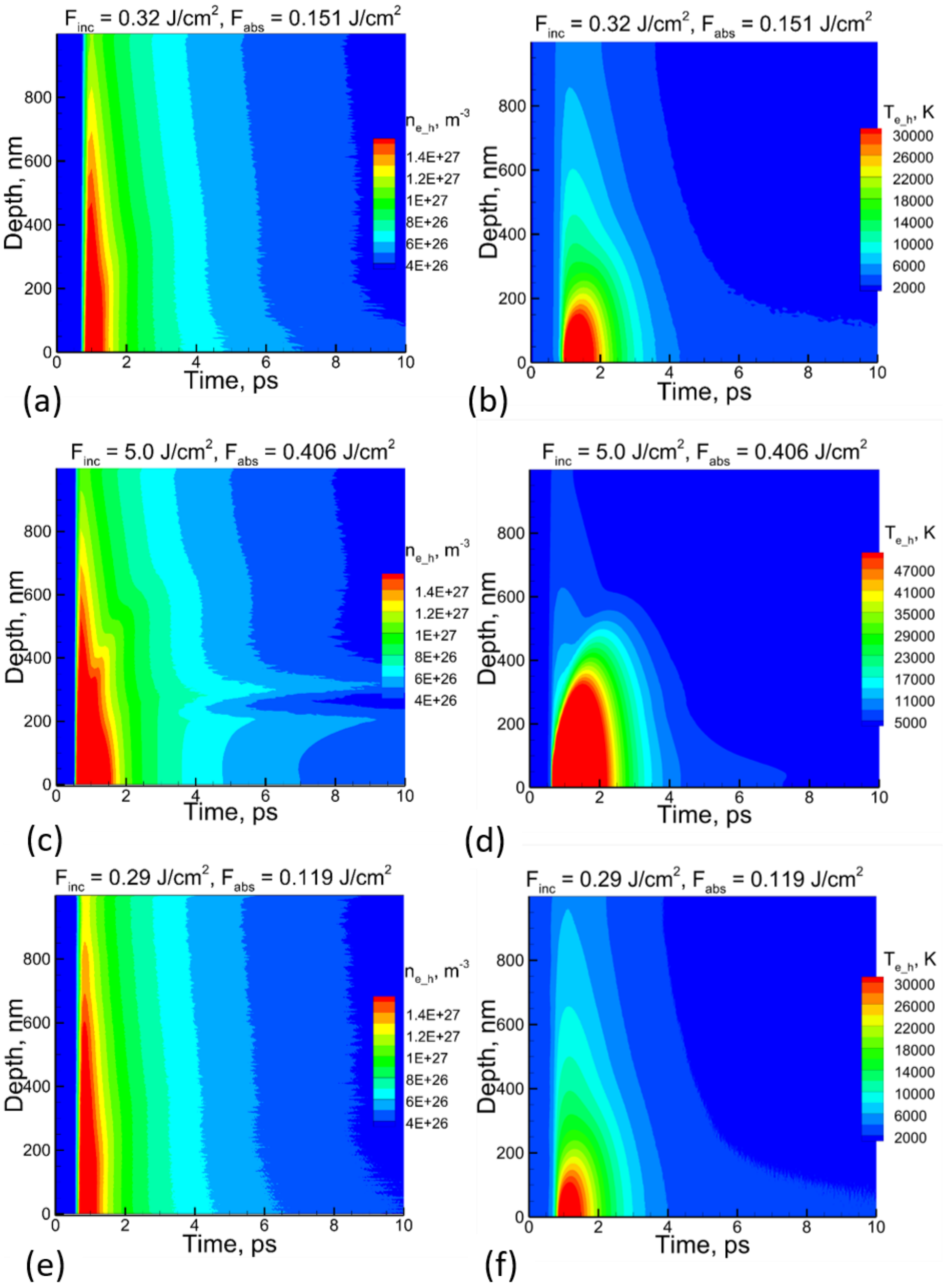

Figure S1. (a) Spatial-temporal field of the free carriers' density and (b) the free carriers' temperature evolutions inside the c-Si upon 270 fs pulse irradiation at 800 nm with the incident fluence of 0.32 J/cm^2. The (c) Spatial-temporal field of the free carriers' density and (d) of the free carriers' temperature evolutions inside the c-Si target after irradiation with a 270 fs pulse at a wavelength of 800 nm and the incident fluence of 5.0 J/cm^2. (e) Spatial-temporal field of the free carriers' density and (f) of the free carriers' temperature evolutions inside the porous (33% by volume) Si target after irradiation with a 270 fs pulse at a wavelength of 800 nm with the incident fluence of 0.29 J/cm^2. In all pictures the laser pulse is directed from the bottom. The dotted line in (c) and (d) indicates the solid-liquid interface. Additional macroscopic data evolution (the electron-hole temperature and the density of free carriers), obtained during the simulation procedure is given in supplementary section.

As it was described in Ref. [64], the conservative form of the nTTM model gives the most accurate results and applicable on a wide range of the energy input (fluence). In order to account on the dynamically changing density of the material due to the process of extension, compression,

ablation, or merely presence of pores, the following scheme of the nTTM model solution can be proposed. We consider the change of materials density in each cell *i* by introducing the multiplier of the relative density $\frac{\rho_i}{\rho_0}$, where ϱ_0 is the initial average density of the sample. The algorithm can be presented as the following steps:

1) Give the initial values of N_e, T_e at the grid points i = 1 : N+1 (see below). It is also convenient to have the values of $F_\xi(\eta_e)$ tabulated.

2) Calculate $\boldsymbol{F_{1/2}(\eta_e)}$ and $\boldsymbol{F_{1/2}(\eta_h)}$ from: $N_e = 2\left[\frac{m_e^* k_b T_e}{2\pi\hbar^2}\right]^{3/2} F_{1/2}(\eta_e)$ and $N_e = 2\left[\frac{m_h^* k_b T_e}{2\pi\hbar^2}\right]^{3/2} F_{1/2}(\eta_h)$

3) Determine the reduced chemical potential value $\boldsymbol{\eta_e}$ from the tabulated data of $F_{1/2}(\eta_e)$. The values of $F_{1/2}$ for the electrons and holes are uniquely connected via their reduced mass (see (2)).

4) Determine the rest of functions: $\boldsymbol{F_{-1/2}(\eta_e), F_0(\eta_e), F_1(\eta_e), F_{3/2}(\eta_e)}$ and the corresponding $\mathbf{H_\xi^\zeta(\eta_e)}$.

5) Repeat 4) for holes $\boldsymbol{F_{1/2}(\eta_h)}$

It is of common practice to have the values of N_e and T_e determined at the grid points and the first derivatives of them (and everything related to fluxes ***J*** and **W**) are at the cells' borders:

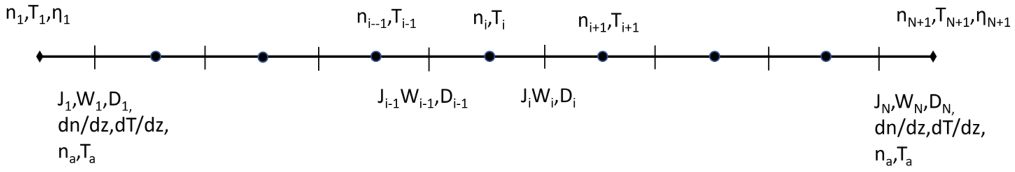

6) Calculate *D*, ***J***, **W**: *j=1 , N*

$$\boldsymbol{D_i} = \frac{k_b T_{e,i+1/2}}{q} \frac{\mu_e^0 \mu_h^0 H_{1/2}^0(\eta_{e,i+1/2}) H_{1/2}^0(\eta_{e,1+1/2})}{\mu_e^0 H_{1/2}^0(\eta_{e,1+1/2}) + \mu_h^0 H_{1/2}^0(\eta_{h,1+1/2})} \left[H_{-1/2}^{1/2}(\eta_{e,i+1/2}) + H_{-1/2}^{1/2}(\eta_{h,i+1/2})\right] * \left(\frac{\boldsymbol{\rho_i \frac{\rho_i}{\rho_0} + \rho_{i+1}\frac{\rho_{i+1}}{\rho_0}}}{\boldsymbol{\rho_i + \rho_{i+1}}}\right)$$

we use the weighted averaging here between *i* and *i+1* cells

$$\overline{\boldsymbol{J_i}} = -D \left\{ \begin{array}{c} \nabla n + \frac{n}{k_b T_e}\left[H_{-1/2}^{1/2}(\eta_e) + H_{-1/2}^{1/2}(\eta_h)\right]^{-1} \nabla E_g + \\ \frac{n}{T_e}\left\{2[H_0^1(\eta_e) + H_0^1(\eta_h)] \Big/ \left[H_{-\frac{1}{2}}^{\frac{1}{2}}(\eta_e) + H_{-\frac{1}{2}}^{\frac{1}{2}}(\eta_h)\right] - \frac{3}{2}\right\} \nabla T_e \end{array} \right\}$$

$$= -D_i \left\{ \begin{array}{c} \frac{n_{i+1} - n_i}{\Delta z} + \frac{n_{i+1/2}}{k_b T_{e,i+1/2}}\left[H_{-1/2}^{1/2}(\eta_{e,i+1/2}) + H_{-1/2}^{1/2}(\eta_{h,1+1/2})\right]^{-1} \frac{E_{g,i+1} - E_{g,i}}{\Delta z} + \\ \frac{n_{i+1/2}}{T_{e,i+1/2}}\left\{2\left[H_0^1(\eta_{e,i+1/2}) + H_0^1(\eta_{h,i+1/2})\right] \Big/ \left[H_{-1/2}^{1/2}(\eta_{e,i+1/2}) + H_{-1/2}^{1/2}(\eta_{h,i+1/2})\right] - \frac{3}{2}\right\} \frac{T_{e,i+1} - T_{e,i}}{\Delta z} \end{array} \right\}$$

$$\overline{\boldsymbol{W_i}} = \left\{E_{g,i+1/2} + 2k_b T_{e,i+1/2}\left[H_0^1(\eta_{e,i+1/2}) + H_0^1(\eta_{h,i+1/2})\right]\right\} \overline{J}_i - \left(\frac{\boldsymbol{\rho_i \frac{\rho_i}{\rho_0} + \rho_{i+1}\frac{\rho_{i+1}}{\rho_0}}}{\boldsymbol{\rho_i + \rho_{i+1}}}\right)(k_e + k_h)\frac{T_{e,i+1} - T_{e,i}}{\Delta z}$$

7) Find the deposited energy solving:

$$\frac{dI(z,t)}{dz} = -\frac{\rho}{\rho_0}\alpha I(z,t) - \frac{\rho}{\rho_0}\beta I^2(z,t) - \frac{\rho}{\rho_0}\Theta_{fca} n I(z,t)$$

with modified Euler method:

Apply $\boldsymbol{I_1(t)} = I_0\sqrt{\frac{\omega}{\pi}}\,\frac{(1-R)}{\tau_{las}}e^{-\omega\frac{(t-t_0)^2}{\tau^2}}$ for the first cell and:

$$I_i^*(z) = I_{i-1} + 0.5dz\frac{\rho_{i-1/2}}{\rho_0}\left(-\alpha_{i-1/2}I_{i-1}(z,t) - \beta_{i-1/2}{I_{i-1}}^2(z,t) - \Theta_{fca_{i-1/2}} n I_{i-1}(z,t)\right)$$

$$\boldsymbol{I_i(z)} = I_{i-1} + dz\frac{\rho_{i-1/2}}{\rho_0}\left(-\alpha_{i-1/2}I_i^*(z,t) - \beta_{i-1/2}I_i^{*2}(z,t) - \Theta_{fca_{i-1/2}} n I_i^*(z,t)\right)$$

8) Perform calculations for new N_e:

$$\frac{\rho}{\rho_0}\frac{\partial N}{\partial t} = \frac{\rho}{\rho_0}\frac{\alpha I(z,t)}{h\nu} + \frac{\rho}{\rho_0}\frac{\beta I^2(z,t)}{2h\nu} - \gamma n^3 + \frac{\rho}{\rho_0}\theta n - \overline{\nabla}\cdot\overline{J}$$

$$\frac{\boldsymbol{N_i^{t+dt}} - N_i^t}{dt} = \frac{\rho_i}{\rho_0}\frac{\alpha_i I_i(z,t)}{h\nu} + \frac{\rho_i}{\rho_0}\frac{\beta_i {I_i}^2(z,t)}{2h\nu} - \frac{\rho_i}{\rho_0}\gamma_i n_i^3 + \frac{\rho_i}{\rho_0}\theta_i n_i - \frac{J_i - J_{i-1}}{dz}$$

9) Calculate $C_{e\text{-}h}$:

$$\boldsymbol{C_{e-h,i}} = \left[\frac{3}{2}k_b n_i\left[H_{1/2}^{3/2}(\eta_{e,i}) + H_{1/2}^{3/2}(\eta_{h,i})\right] + \frac{3}{2}n_i k_b T_{e,i}\left[1 - H_{1/2}^{3/2}(\eta_{e,i})H_{1/2}^{-1/2}(\eta_{e,i})\right] + \frac{3}{2}n_i k_b T_{e,i}\left[1 - H_{1/2}^{3/2}(\eta_{h,i})H_{1/2}^{-1/2}(\eta_{h,i})\right] + n_i\frac{\partial E_{g,i}}{\partial T_{e,i}}\right]$$

10) Calculate new $U_{e\text{-}h}$:

$$\frac{\partial U_{e-h}(n,T_e)}{\partial t} = \frac{\rho}{\rho_0}\alpha I(z,t) + \frac{\rho}{\rho_0}\beta I^2(z,t) + \frac{\rho}{\rho_0}\Theta_{fca} n_i I(z,t) - \overline{\nabla}\cdot\overline{W} - \frac{\rho}{\rho_0}\frac{C_{e-h}}{\tau_{e-h}}(T_e - T_l)$$

$$\frac{\boldsymbol{U_i^{t+dt}} - U_i^t}{dt} = \frac{\rho_i}{\rho_0}\alpha_i I_i(z,t) + \frac{\rho_i}{\rho_0}\beta_i {I_i}^2(z,t) + \frac{\rho_i}{\rho_0}\Theta_{fca_i} n_i I_i(z,t) - \frac{W_i - W_{i-1}}{dz} - \frac{\rho_i}{\rho_0}\frac{C_{e-h,i}}{\tau_{e-h,i}}\left(T_{e,i} - T_{l,i}\right)$$

11) The equation for lattice temperature T_l can be solved as the following:

$$\frac{\rho}{\rho_0}C_l\frac{\partial T_l}{\partial t} = \overline{\nabla}\cdot k_l\nabla T_l + \frac{\rho}{\rho_0}\frac{C_{e-h}}{\tau_{e-h}}(T_e - T_l)$$

$$\frac{\rho_i}{\rho_0}C_l\frac{\boldsymbol{T_{l,i}^{t+dt}} - T_{l,i}^t}{dt} = \frac{\frac{\rho_{i+1/2}}{\rho_0}k_{l,i+1/2}\left(T_{l,i+1} - T_{l,i}\right) - \frac{\rho_{i-1/2}}{\rho_0}k_{l,i-1/2}\left(T_{l,i} - T_{l,i-1}\right)}{dz^2} + \frac{\rho_i}{\rho_0}\frac{C_{e-h,i}}{\tau_{e-h,i}}\left(T_{e,i} - T_{l,i}\right)$$

12) Solve: $U_{e-h}(n,T_e) = n\left\{E_g + \frac{3}{2}k_b T_e\left[H_{1/2}^{3/2}(\eta_e) + H_{1/2}^{3/2}(\eta_h)\right]\right\}$ as a transcendent equation subject to new value of temperature T_e. The necessary date can be preliminary tabulated.

First, we find new value for the energy gap: $E_g^{t+dt}\left(n_i^{t+dt}, T_{e,i}^t, T_{l,i}^{t+dt}\right)$

Then:

$$\frac{U_{e-h,i}^{t+dt} - n_i^{t+dt} E_g^{t+dt}}{n_i^{t+dt} \frac{3}{2} k_b} = T_{e,i}^{t+dt} \left[H_{1/2}^{3/2}(\eta_{e,i}^{t+dt}) + H_{1/2}^{3/2}(\eta_{h,i}^{t+dt}) \right]$$

We use $n = 2\left[\frac{m_e^* k_b T_e}{2\pi\hbar^2}\right]^{3/2} F_{1/2}(\eta_e) \quad for \quad T_e = \left(\frac{n}{2F_{1/2}(\eta_e)}\right)^{\frac{2}{3}} \frac{2\pi\hbar^2}{m_e^* k_b}$

$$\frac{U_{e-h,i}^{t+dt} - n_i^{t+dt} E_g^{t+dt}}{n_i^{t+dt} \frac{3}{2} k_b} = \left(\frac{n_i^{t+dt}}{2F_{1/2}(\eta_{e,i}^{t+dt})}\right)^{2/3} \frac{2\pi\hbar^2}{m_e^* k_b} \left[H_{1/2}^{3/2}(\eta_{e,i}^{t+dt}) + H_{1/2}^{3/2}(\eta_{h,i}^{t+dt}) \right]$$

Reorganizing this expression, we arrive to the one, which can be solved with respect to new values of $\eta_{e,i}^{t+dt}$from the tabulated data:

$$\frac{U_{e-h,i}^{t+dt} - n_i^{t+dt} E_g^{t+dt}}{\left(n_i^{t+dt}\right)^{5/3} 3\sqrt{2}\pi\hbar^2 / m_e^*} = \left[\frac{\boldsymbol{F_{3/2_i}(\eta_{e,i}^{t+dt})}}{\left(\boldsymbol{F_{1/2_i}(\eta_{e,i}^{t+dt})}\right)^{5/3}} + \frac{\boldsymbol{F_{3/2_i}(\eta_{h,i}^{t+dt})}}{\left(\boldsymbol{F_{1/2_i}(\eta_{e,i}^{t+dt})}\right)^{2/3} \boldsymbol{F_{1/2,_i}(\eta_{h,i}^{t+dt})}} \right]$$

13) Extract new value for $\boldsymbol{\eta_{e,i}^{t+dt}}$

14) Find the new values for $\boldsymbol{T_{e,i}^{t+dt}} = \left(\frac{n_i^{t+dt}}{2F_{1/2}(\eta_{e,i}^{t+dt})}\right)^{2/3} \frac{2\pi\hbar^2}{m_e^* k_b}$

15) Overwrite the arrays of temperature: ***T_e[1:N+1] = T_e^{new}[1:N+1]***

16) Repeat the steps 6) - 15) until reaching the required length of simulation.
